# Fast Inhibition of Glutamate-Activated Currents by Caffeine

**DOI:** 10.1371/journal.pone.0003155

**Published:** 2008-09-10

**Authors:** Nicholas P. Vyleta, Stephen M. Smith

**Affiliations:** Division of Pulmonary & Critical Care Medicine, Oregon Health & Science University, Portland, Oregon, United States of America; Tel Aviv University, Israel

## Abstract

**Background:**

Caffeine stimulates calcium-induced calcium release (CICR) in many cell types. In neurons, caffeine stimulates CICR presynaptically and thus modulates neurotransmitter release.

**Methodology/Principal Findings:**

Using the whole-cell patch-clamp technique we found that caffeine (20 mM) reversibly increased the frequency and decreased the amplitude of miniature excitatory postsynaptic currents (mEPSCs) in neocortical neurons. The increase in mEPSC frequency is consistent with a presynaptic mechanism. Caffeine also reduced exogenously applied glutamate-activated currents, confirming a separate postsynaptic action. This inhibition developed in tens of milliseconds, consistent with block of channel currents. Caffeine (20 mM) did not reduce currents activated by exogenous NMDA, indicating that caffeine block is specific to non-NMDA type glutamate receptors.

**Conclusions/Significance:**

Caffeine-induced inhibition of mEPSC amplitude occurs through postsynaptic block of non-NMDA type ionotropic glutamate receptors. Caffeine thus has both pre and postsynaptic sites of action at excitatory synapses.

## Introduction

The popular stimulant, caffeine, modulates intracellular calcium signaling in many cell types [1]. The ryanodine receptor (RyR) is one target for caffeine. Millimolar concentrations (5–20 mM) of caffeine stimulate calcium-induced calcium release (CICR) from intracellular stores. Caffeine sensitizes RyR so that low nanomolar concentrations of cytosolic calcium activate RyR leading to calcium efflux into the cytoplasm [2]. In addition, caffeine also acts on the other primary endoplasmic reticulum calcium release channel, the inositol triphosphate receptor (IP_3_R), over the same concentration range. Caffeine inhibited IP_3_R single-channel openings with a half-maximal inhibition of 1.6 mM [3]. Therefore, depending on the relative densities of RyR and IP_3_R in a particular cell, caffeine can either stimulate or block calcium release from intracellular stores.

Caffeine has been used extensively to study calcium signaling in neurons. Caffeine increases spontaneous glutamate release from nerve terminals [4] and can induce presynaptic long-term potentiation [5]. Synaptically activated AMPA receptor-mediated CICR has also been described using caffeine [6].

Here, we demonstrate that caffeine decreases the size of miniature excitatory postsynaptic currents (mEPSCs) recorded from neocortical neurons by directly inhibiting postsynaptic glutamate receptor currents. Consistent with other reports, we also demonstrate an increase in mEPSC frequency indicating a caffeine-induced presynaptic increase in glutamate release. Thus, caffeine modulation of glutamatergic transmission involves both pre and postsynaptic targets.

## Results

### Caffeine decreases mEPSC size in neocortical neurons

**Figure 1:**
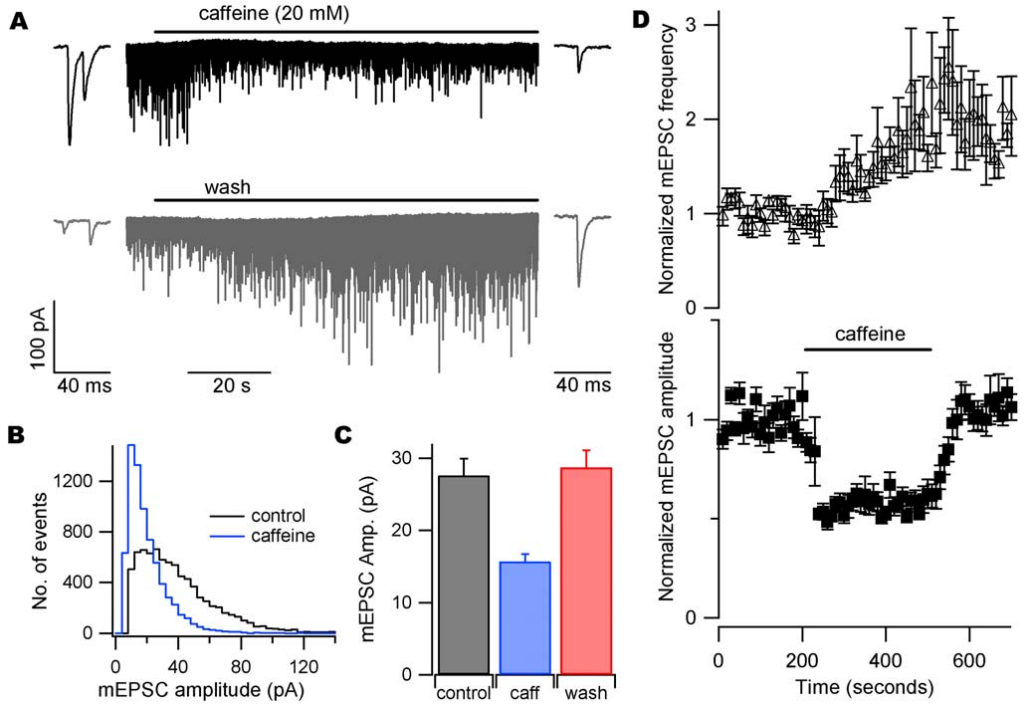
Caffeine reversibly decreases mEPSC amplitude. (A) Recording of mEPSCs in whole-cell voltage-clamp in the presence of TTX (1 µM). Caffeine (20 mM) caused a clear reversible decrease in mEPSC size. The fast rise and exponential fall of synaptic currents was clear throughout recording (insets). (B) Amplitude histograms of events in A 200 seconds immediately before caffeine (black) and during the last 200 seconds of caffeine application (blue). (C) Average mEPSC amplitudes for seven cells before (black), during (blue), and after (red) caffeine application. (D) Normalized average diary plots of mEPSC frequency (top) and amplitude (bottom), n = 7. Bin size = 10 seconds. Each point is normalized to the average of 200 seconds of data recorded before drug application.

We examined the effects of caffeine (20 mM) on mEPSCs recorded from neocortical neurons. Sodium channels were blocked by tetrodotoxin (TTX) and spontaneous synaptic events recorded at −70 mV. Caffeine was applied for 300 seconds. The amplitude of mEPSCs decreased during the first 100 seconds of caffeine application (Fig. 1A). The insets show representative mEPSCs from the beginning and end of their respective traces on an expanded time scale. The inhibition of mEPSC amplitude was reversible (Fig. 1A). Amplitude histograms of all events during 200 seconds of recording directly before caffeine application and all events recorded during the last 200 seconds of caffeine application confirmed this inhibition (Fig. 1B). Average mEPSC amplitude is shown for seven cells before, during, and after caffeine application (Fig. 1C). Average mEPSC amplitude was 27.6±2.3 pA before and 15.7±1.0 pA during caffeine application (P<0.001; n = 7), demonstrating a 43±4% inhibition by caffeine. Miniature EPSC amplitude was reversibly decreased by caffeine in all seven cells. Fig. 1D shows average normalized diary plots of mEPSC frequency (top) and amplitude (bottom) for seven cells. Caffeine increased mEPSC frequency in all seven cells, consistent with previous work [4]. The increase in mEPSC frequency was slower than the decrease in amplitude, suggesting different sites of action. These data confirm that caffeine increases mEPSC frequency in neocortical neurons, and demonstrate a reduction in mEPSC amplitude not previously described.

### Caffeine blocks currents activated by exogenous glutamate

**Figure 2:**
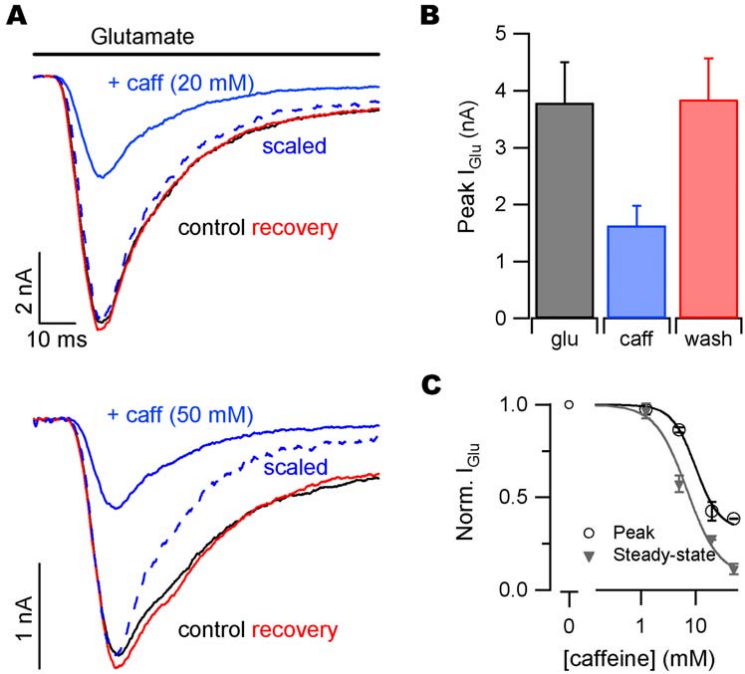
Caffeine inhibits postsynaptic glutamate-activated currents (I_glu_). (A) Glutamate (1 mM) was applied to neurons in whole-cell voltage clamp in the presence of TTX. Individual traces from a representative recording of I_glu_ before (black), during (blue) and after (red) the addition of 20 mM (top) or 50 mM (bottom) caffeine. (B) Average peak I_glu_ for 5 consecutive applications of glutamate (3 second interval) before, during, and after 20 mM caffeine application. (C) Normalized peak (open circles) and steady-state (closed triangles) I_glu_ for 1.25, 5, 20, and 50 mM caffeine. Values normalized to average peak or steady-state I_glu_ before caffeine for each recording. Curves represent Equation 1.

We next asked whether caffeine exerted its effects on mEPSC amplitude by a presynaptic or postsynaptic mechanism. We applied glutamate (1 mM) to test for a direct postsynaptic effect of caffeine on glutamate-activated currents (I_glu_). Glutamate was applied with a piezoelectric-controlled perfusion device to neurons voltage-clamped at −70 mV. Glutamate was applied for 100 milliseconds and co-application with caffeine reversibly decreased peak I_glu_ amplitude (Fig. 2A). In these experiments glutamate applications were made after perfusion with caffeine containing solutions for >30 seconds (top 20 mM and bottom 50 mM). Bar graphs show the average results from glutamate application in the presence of 20 mM caffeine (Fig. 2B). Glutamate-activated currents were 3.79±0.71 nA and reduced to1.63±0.35 nA in the presence of caffeine (n = 7; P = 0.002). Currents recovered after caffeine wash to 3.84±0.72 nA (P = 0.10 compared to control). Thus, caffeine (20 mM) reversibly reduced the magnitude of I_glu_ by 57±5%. The action of caffeine was dose-dependent (Fig. 2C). Inhibition of I_glu_ by caffeine increased during the application (scaled traces, Fig. 2A). Consequently the steady state concentration-effect relationship was left-shifted relative to that for peak glutamate-activated currents (Fig. 2C). Curves represent the equation:
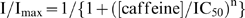
(1)where IC_50_ was 7 mM and 10 mM for inhibition of the steady-state and peak glutamate-activated currents, respectively.

### Caffeine blocks glutamate-activated currents rapidly

**Figure 3:**
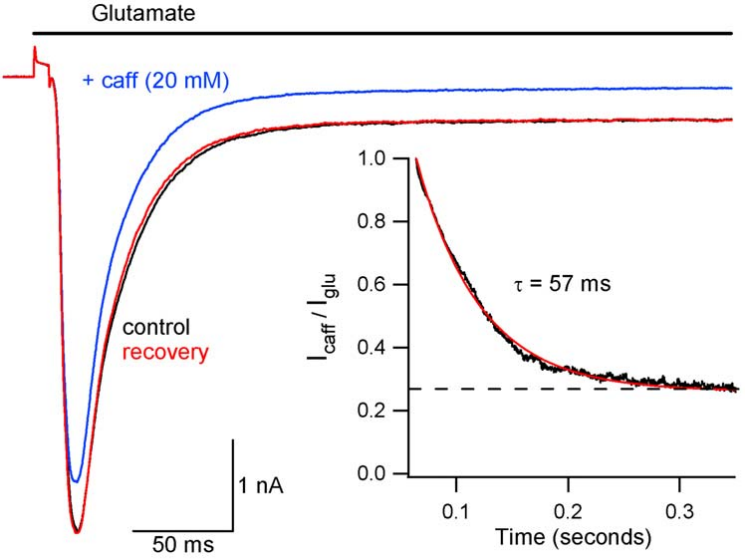
Caffeine rapidly inhibits glutamate-activated currents. After control recording of I_glu_ (black), glutamate was applied simultaneously with 20 mM caffeine (I_caff_; blue). Recovery is shown in red. Traces are averages of 5 consecutive applications of glutamate or glutamate plus caffeine (3 second interval). Inset: I_caff_/I_glu_ plotted versus time for representative cell (black) to show time course of inhibition of I_glu_ by caffeine. Calculation was started 10 ms after start of glutamate application, shortly before peak of I_glu_. Decay was fit to a single exponential (red curve). Steady-state inhibition of I_glu_ (dashed line) is 0.27 for this recording.

In previous experiments (Fig. 2) glutamate was applied after >30 seconds of caffeine application. We next measured the kinetics of caffeine block of glutamate-activated currents by applying caffeine (20 mM) simultaneously with glutamate (1 mM). Fig. 3 shows I_glu_ with and without caffeine. Each trace is the average I_glu_ elicited by 5 applications of glutamate recorded with a 0.33 Hz duty cycle. Traces did not change substantially between applications. Caffeine block of I_glu_ developed during a 500 ms co-application of glutamate and caffeine. Inhibition is absent during the initial rising phase of I_glu_, partial during the peak, and reaches steady-state by ∼200 ms. The effect was reversible. The time course of caffeine block was quantified by dividing the glutamate-activated current in the presence of caffeine (I_caff_) by the previous current elicited by glutamate alone. This ratio (I_caff_/I_glu_) is plotted as a function of time for the representative recording starting 10 ms after initiation of glutamate application (black, Fig. 3 inset). This decay was fit with a single exponential function with a time constant of 57 ms. The average time constant for inhibition was 52±12 ms (n = 3). The dashed line represents the steady-state inhibition of I_glu_ and crosses the y-axis at 0.27 for this recording. The average steady-state value for I_caff_/I_glu_ was 0.29±0.02, which agrees well with the inhibition of steady-state I_glu_ in the presence of steady-state caffeine concentration (0.28, Fig. 2C). Thus, caffeine action is rapid, and I_glu_ blockade develops during glutamate application (Fig. 2). These data demonstrate that caffeine inhibits glutamate receptor current by a fast mechanism, consistent with simple channel block but not consistent with more complex forms of regulation such as changes in receptor expression.

### Caffeine-mediated block of glutamate receptor currents is specific for non-NMDA type ionotropic glutamate receptors

**Figure 4:**
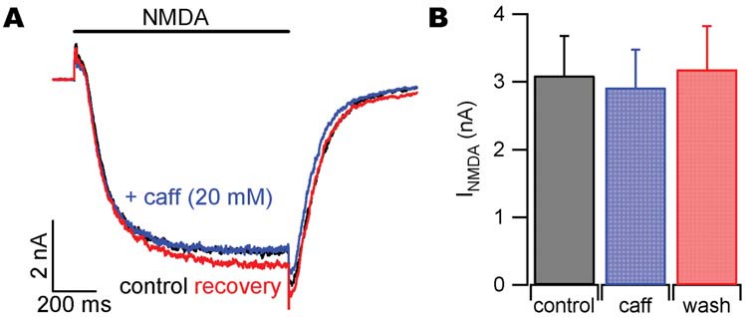
Caffeine does not inhibit postsynaptic NMDA-receptor mediated currents (I_NMDA_). (A) NMDA (20 µM) was applied to neurons in whole-cell voltage clamp in the presence of glycine and TTX, and zero extracellular Mg^2+^. Individual traces from a representative recording of I_NMDA_ before (black), during (blue) and after (red) the addition of 20 mM caffeine. (B) Average peak I_NMDA_ for single applications of NMDA before, during, and after 20 mM caffeine application (120 second interval).

Both AMPA and NMDA-activated currents are activated by glutamate application to neocortical neurons. The mEPSCs in neocortical neurons were blocked by CNQX and thus are mediated by non-NMDA receptors (data not shown). To test if caffeine also blocked NMDA-activated currents we applied NMDA (20 µM) in the absence and presence of caffeine. NMDA-mediated currents (I_NMDA_) were evoked in the presence of 2.5 µM glycine and in the absence of extracellular magnesium every 2 minutes. I_NMDA_ were minimally affected by the application of 20 mM caffeine (Fig. 4A). Average data for NMDA application to six cells is shown in Fig. 4B. I_NMDA_ was 3.09±0.59 nA before and 2.92±0.56 nA in the presence of 20 mM caffeine (n = 6; P = 0.21). After caffeine wash, I_NMDA_ recovered to 3.18±0.64 (P = 0.39 compared to control). In addition, glutamate (200 µM) was applied to neurons in the presence of CNQX (25 µM) and glycine (2.5 µM) in the absence of magnesium. These NMDA receptor-mediated currents did not change in the presence of 20 mM caffeine (n = 2, data not shown). Our results confirm that caffeine-mediated inhibition of mEPSC amplitude and I_glu_ are not mediated by NMDA receptors.

## Discussion

Caffeine modulates intracellular calcium and can stimulate neurotransmitter release from nerve terminals [4]. We determined that the frequency of spontaneous transmitter release was increased in recordings from neocortical neurons. Surprisingly, caffeine also produced an accompanying decrease in the amplitude of quantal events. Caffeine inhibited currents activated by direct application of glutamate to cortical neurons, confirming a postsynaptic site of action. This unexpected form of inhibition developed over tens of milliseconds and was independent of NMDA receptors, consistent with non-NMDA receptor block.

### Caffeine modulates synaptic transmission by both pre- and postsynaptic mechanisms

We found that caffeine increased the frequency of mEPSCs, consistent with a previous report [4] (Fig. 1). A change in the frequency of spontaneous neurotransmitter release events is a clear indicator of a presynaptic change in the probability of vesicle fusion. A change in quantal size could result from either pre or postsynaptic mechanisms [7] – variation in mEPSC size was recently shown to depend on vesicular glutamate concentration [8]. We postulated that caffeine-mediated changes in mEPSC amplitude and frequency (Fig. 1) both resulted from presynaptic modulation. However, the distinct time courses of both changes pointed to different mechanisms of action (Fig. 1D). Caffeine inhibited currents activated by exogenous glutamate and quantal events by a similar amount confirming a postsynaptic action. We postulated two mechanisms by which caffeine mediated these effects: by directly blocking the glutamate activated channels or by reducing postsynaptic glutamate receptor density. However, decreases in receptor density due to endocytosis of AMPA receptors (AMPARs) is probably too slow to explain this effect since it occurs over tens of seconds[9]. In contrast, caffeine mediated block of I_glu_ was rapid, developing in tens of milliseconds (Fig. 3), or ∼1000 times faster than endocytosis of receptors. These data support the proposal that caffeine decreases mEPSC amplitude by direct postsynaptic block of glutamate-activated channels.

Caffeine mediated inhibition of mEPSCs was slightly smaller than inhibition of peak glutamate-activated currents (43% vs. 57%, respectively). While this observation could be due to extrasynaptic glutamate receptors being more sensitive to caffeine, we feel it more likely reflects a sampling bias caused by selecting mEPSCs based on an amplitude threshold. Our use of a threshold means that proportionately more of the smaller mEPSCs in caffeine failed to reach detection reducing the amount of caffeine-mediated inhibition. This effect is evident in the mEPSC amplitude histogram (Fig. 1B).

### Caffeine modulation of glutamate signaling

Recently it was reported that caffeine blocks CICR triggered by calcium influx through calcium permeable AMPARs [6]. Glutamate or AMPA-induced increases in intracellular calcium concentration were decreased in the presence of caffeine. This result was interpreted as a caffeine-induced depletion of ryanodine-sensitive calcium store calcium. Our results provide an additional interpretation to their data: that caffeine directly blocks AMPAR-mediated calcium currents responsible for CICR. Morton-Jones et al. provide strong additional evidence, however, that glutamate-activated intracellular calcium increases are mediated by CICR by blocking them with the RyR blocker ryanodine.

In contrast to our findings, caffeine (10 mM) has been reported to increase mEPSC amplitude in recordings from barrel cortex slices [10]. The authors showed an increase in median mEPSC amplitude from ∼2.5 to 4.5 pA in the presence of caffeine. These small mEPSCs would have been difficult to distinguish from baseline fluctuations by automated analysis. In fact, the median event size represented only 3–4 channel openings (using AMPA-activated channel conductance∼10 pS [11] and 70 mV driving voltage). It is possible that these small events reflected baseline channel activity that was mischaracterized as representing mEPSCs. If caffeine application increased mEPSC frequency, and these mEPSCs were larger than the baseline events, this would have resulted in an apparent increase in mEPSC size. The mEPSCs we recorded were much larger (19 to 39 pA; Fig. 1C) and clearly distinguished from baseline activity since our detection threshold was ∼5 pA. Thus the increased signal-noise ratio in our experiments provides strong evidence that caffeine inhibits mEPSC amplitude.

### Caffeine actions *in vivo*

The widespread use of caffeine begs the question whether glutamate receptor blockade may occur in humans. Caffeine concentration in blood reaches only 60 µM after ingestion of the equivalent of 4 cups of coffee [12]. The blood-brain barrier is readily permeable to caffeine, so the concentration in the brain is close to that in the blood [13]. Since caffeine inhibits glutamate receptors with an apparent IC_50_ of ∼10 mM (Fig. 2C, equation 1) we suggest that ingested caffeine is unlikely to have any effect on ionotropic glutamate receptors. Instead caffeine likely produces stimulatory effects in humans through its potent antagonism of the adenosine receptor (IC_50_ 50–100 µM) [14]. Adenosine-mediated activation of A1 adenosine receptors on nerve terminals impairs synaptic transmission by inhibiting voltage-gated calcium channels [15]. Thus, one mechanism by which normal physiological levels of caffeine stimulates synaptic transmission is by inhibiting the adenosine binding, relieving the inhibition of voltage-gated calcium channels and producing higher probability of release of neurotransmitter.

However, it is possible that excess use of caffeine tablets may lead to brain levels at which glutamate-activated channel modulation occurs. For instance, acute caffeine toxicity has been blamed for the death of a 22-yr old woman who experienced serum caffeine levels of ∼8 mM following overdose with diet pills [16].

### Conclusions

We have demonstrated a novel action of caffeine on excitatory transmission in the central nervous system. We show that, in addition to increasing the probability of spontaneous release of neurotransmitter, caffeine inhibits postsynaptic AMPA-type glutamate-activated channels. This occurred at caffeine concentrations regularly used for studying intracellular calcium signaling. Furthermore, our results are consistent with the hypothesis that caffeine-mediated glutamate receptor blockade may only occur under extreme conditions of toxicity.

## Methods

### Neuronal preparation

Neocortical neurons were isolated from P1-2 mouse pups. All animal procedures were approved by OHSU I.A.C.U.C. in accordance with the U.S. Public Health Service Policy on Humane Care and Use of Laboratory Animals and the N.I.H. Guide for the Care and Use of Laboratory Animals. Animals were deeply anesthetized with isoflurane before decapitation and removal of cortices. Cortices were then incubated in trypsin and DNAse and then dissociated with a heat polished pipette. Dispersed cells were cultured in MEM plus 5% FBS on glass coverslips. ARAC (4 µM final concentration) was added 48 hours after plating to limit glial division. Cells were used after at least 14 days in culture. Eight different neuronal culture preparations from eight different mice were used for these experiments.

### Electrophysiological recordings

Cells were visualized with an Olympus IX70 inverted microscope. Recordings were made in whole-cell voltage clamp. Holding potential was −75 mV. Extracellular solutions contained (in mM) 150 NaCl, 4 KCl, 10 HEPES, 10 glucose, pH 7.35. NaCl was substituted with either caffeine or sucrose to maintain osmolarity. Recordings of mEPSCs were made in the presence of TTX (1 µM) and bicuculline (10 µM) to block Na channels and GABA-activated currents, respectively. TTX was also used during glutamate application experiments. Intracellular solution consisted of (in mM) 140 K^+^ gluconate, 9 EGTA, 10 HEPES, 4 MgCl_2_, 1 CaCl_2_, 4 NaATP, 0.3 NaGTP, 1.4 phosphocreatine, pH 7.2. Electrode tips had final resistances of 3–6 MΩ. Currents were recorded with a HEKA EPC9/2 amplifier. For mEPSC recordings, currents were filtered at 1 kHz using a Bessel filter and sampled at 10 kHz. For recordings of currents evoked by applied glutamate, currents were filtered at 3 kHz and sampled at 20 kHz. Series resistance (R_s_) was monitored, and recordings were discarded if R_s_ changed by more than 10% during recording. For glutamate-application experiments, R_s_ was usually compensated by ∼70%. We estimate that in a typical experiment our inhibition of I_glu_ by caffeine (20 mM) is underestimated by ∼9% [17].

### Solution application

For mEPSC recordings, solutions were bath applied through a perfusion pipette placed ∼1 mm from the patch pipette tip. Local solution equilibration occurred in <10 seconds as measured by open-tip conductance changes. For fast application of glutamate, a custom-built piezoelectric-driven perfusion system was used. A piece of theta glass containing four small perfusion tubes (two in each barrel) was mounted to a piezoelectric bimorph (Piezo Systems, Inc., product # T234-A4CL-203X) which was mounted to a plastic rod controlled by a micromanipulator. A high-voltage stimulus isolator (World Precision Instruments, Sarasota, Florida, product # A360D or A365D) was used to stimulate the bimorph. A TTL pulse supplied by one of the digital-to-analog outputs on the EPC9/2 was used to drive the stimulus isolator. Theta glass was pulled and broken to a tip diameter of approximately 300 microns. A smooth break of barrels and septum was achieved to minimize solution mixing at tip. During a whole-cell voltage clamp recording, the perfusion tip was located so that zero steady-state glutamate activated current was detected. Stimulation of the bimorph moved the solution interface across the neuron. Time of perfusion change to whole neuron was estimated with open-tip conductance measurements with electrode tip at a distance from the theta tube characteristic of a cell soma. Total solution change in this configuration occurred in ∼5 ms.

### Analysis

Data were acquired on a PIII computer and analyzed with IgorPro (Wavemetrics, Lake Oswego, OR) and Minianalysis (Syanptosoft, Decatur, GA) software. For all experiments, statistical significance was determined using Student's t-test as appropriate (Microsoft EXCEL, Richmond, WA). Averaged data values are reported as means±SEM.
